# Classification of patients with Alzheimer’s disease using the arterial pulse spectrum and a multilayer-perceptron analysis

**DOI:** 10.1038/s41598-021-87903-7

**Published:** 2021-04-26

**Authors:** Shun-Ku Lin, Hsin Hsiu, Hsi-Sheng Chen, Chang-Jen Yang

**Affiliations:** 1 Institute of Public Health, National Yang-Ming University, Taipei, Taiwan; 2 Department of Chinese Medicine, Taipei City Hospital, Renai Branch, Taipei, Taiwan; 3 General Education Center, University of Taipei, Taipei, Taiwan; 4 Graduate Institute of Biomedical Engineering, National Taiwan University of Science and Technology, No. 43, Section 4, Keelung Road, Taipei, 10607 Taiwan; 5 Biomedical Engineering Research Center, National Defense Medical Center, Taipei, Taiwan

**Keywords:** Health care, Biomedical engineering

## Abstract

Cerebrovascular atherosclerosis has been identified as a prominent pathological feature of Alzheimer’s disease (AD); the link between vessel pathology and AD risk may also extend to extracranial arteries. This study aimed to determine the effectiveness of using arterial pulse-wave measurements and multilayer perceptron (MLP) analysis in distinguishing between AD and control subjects. Radial blood pressure waveform (BPW) and finger photoplethysmography signals were measured noninvasively for 3 min in 87 AD patients and 74 control subjects. The 5-layer MLP algorithm employed evaluated the following 40 harmonic pulse indices: amplitude proportion and its coefficient of variation, and phase angle and its standard deviation. The BPW indices differed significantly between the AD patients (6247 pulses) and control subjects (6626 pulses). Significant intergroup differences were found between mild, moderate, and severe AD (defined by Mini-Mental-State-Examination scores). The hold-out test results indicated an accuracy of 82.86%, a specificity of 92.31%, and a 0.83 AUC of ROC curve when using the MLP-based classification between AD and Control. The identified differences can be partly attributed to AD-induced changes in vascular elastic properties. The present findings may be meaningful in facilitating the development of a noninvasive, rapid, inexpensive, and objective method for detecting and monitoring the AD status.

## Introduction

Alzheimer’s disease (AD) is the most common cause of age-related cognitive impairment, and is now the fifth leading cause of death worldwide, making it a leading health challenge^1,2^. The ability to detect AD in its early stages is therefore becoming an urgent task. Many of the existing methods for diagnosing AD are either invasive or expensive, such as lumbar puncture, PET, and MRI. For example, the ratio between the concentrations of total tau protein and Aβ(1–42) in the cerebrospinal fluid has been used as an early biomarker of AD^3^. However, it remains crucial to develop accurate, risk-free alternative methods for diagnosing AD.

AD has traditionally been considered a neurodegenerative disease affecting neurons, but alterations of the cerebral macrovasculature and microvasculature are also present^1^. The brain is an organ whose structural and functional integrity is critically dependent on its blood supply. The development of atherosclerosis in cerebral vessels may compromise the oxygen and energy supply to the brain due to hypoperfusion, with deleterious neurological consequences^2^.

Cerebrovascular atherosclerosis has been identified as a prominent pathological feature of AD^2^. These vascular changes include functional and structural alterations, such as reduced cerebral perfusion and an impaired ability of the cerebral circulation to supply energy substrates and oxygen to active brain regions^2^. These vascular alterations are suggested to play a role in the neuronal dysfunction and damage underlying this type of dementia^1^. Presymptomatic individuals at risk of AD exhibit cerebrovascular dysfunction, reduced cerebral blood flow (CBF), and altered permeability of the blood–brain barrier; these raise the possibility that cerebrovascular dysfunction is a presymptomatic correlate of AD pathology^1^. This hypothesis can be supported by transgenic mice with amyloid accumulation reportedly exhibiting profound disruption of neurovascular regulation before the expression of cognitive deficits and amyloid plaques^4^.

The link between atherosclerosis and the development of AD is related to not only intracranial vessels but also extracranial arteries^2^. For example, atherosclerosis has been noted in both the carotid and femoral arteries, while coronary artery calcifications have also been suggested to be linked to a higher probability of dementia in elderly individuals^5^. It has been suggested that systemic atherosclerosis—together with large-artery stiffening and a procoagulant state in AD—may cause damage to the cerebral vasculature and further contribute to alterations in the CBF. Such vascular alterations outside the brain have also been suggested to play a role in AD^2^.

Blood pressure (BP) waveform (BPW) is determined by the interaction of the heart blood pumping and the arterial tree; it can provide information about arterial wall integrity and arterial stiffness^6,7^. Increased arterial stiffness may be involved in the pathogenesis of cardiovascular disease by a number of mechanisms^8^. Since several diseases can induce changes in the arterial stiffness, and thereby affect the amplitude and speed of arterial pulse-wave transmission and thus change the arterial pulse waveform, measurements and time-domain analyses of the pulse waveform may aid the monitoring of the disease condition. Different parameters trying to capture changes in pulse wave morphology and relating them to physiological changes have been proposed and applied in clinical studies^9^. For example, pulse wave velocity and the augmentation index calculated from the time-domain pulse waveform are indicators of arterial stiffness^9,10^. Increased arterial stiffness has been associated with an increased risk of developing cardiovascular diseases and all-cause mortality^11,12^. Aging, hypertension, peripheral cardiovascular disease, hypercholesterolaemia, and diabetes were noted to influence arterial properties and modify the amplitude and timing of forward and backward waves^13,14^. Among the time-domain indices, the augmentation index is known to be associated with the presence and severity of coronary artery disease^15,16^. Both pulse wave velocity and augmentation index correlate with cardiovascular risk; many treatments that reduce BP lead to a reduction in pulse wave velocity and augmentation index^15^.

In the frequency domain, differences have also been found in the BPW and photoplethysmography (PPG) pulse indices between control subjects and patients with various kinds of diseases, including metabolic syndrome^17^, polycystic-ovary syndrome^18^, breast cancer^19^, and frozen shoulder^20^. For cerebrovascular disease, the dynamics of cerebral autoregulation may be assessed by comparing continuous measurements of the CBF with simultaneous measurements of spontaneous fluctuations in the BP^21^. Frequency-domain analysis of the BPW can also be used to monitor the distribution of the blood supply, and such analyses have identified significant differences in BPW indices between the stroke and contralateral sides in individual patients^22^. This finding illustrates that analyzing the pulse waveform can help to monitor changes in the vascular properties of the cerebrovascular system.

Modern machine-learning techniques are already widely employed in biological domains such as genomics, proteomics, microarrays and systems biology^23^, and have been used to estimate the cardiovascular indices or to predict cardiovascular health condition. For example, combinations of statistical, spectral, and nonlinear features of short-term heart rate variability have been used (discriminant analysis, support vector machine, *k*-nearest neighbors, Naive Bayes, and decision trees were used) to aid the diagnosis of arterial hypertension^24^. Pulse transit and arrival timings derived from PPG signals have been used as features to estimate aortic pulse wave velocity, diastolic BP, systolic BP and stroke volume (Gaussian process regression was used)^25^. Machine-learning approaches have also been applied to the pulse waveform indices. Classification algorithms (random forest, BayesNet, J48 decision tree and RIPPER rule-based induction were used) using features extracted from BPW have been reported to efficiently discriminate between hypertensive and healthy control subjects^6^. An automatic method (*k*-nearest neighbours and support vector machine were used) to classify the BPW signals and noise segments was developed by using a pool of 37 features (including amplitude features, time domain statistics, wavelet features, cross-correlation features and frequency domain statistic)^7^. Machine-learning techniques have been reported to be applied to the differential diagnosis of dementia, such as neuroimaging, cognitive assessment data and speech analysis^23,26,27^. Frequently used machine-learning techniques include support vector machines, decision trees, Bayesian networks, and artificial neural networks^23^. For example, an effective differentiation was reported between AD and frontotemporal dementia based on MRI scans and machine learning (support vector machine was used)^28^, while neuropsychological testing as a predictor for a machine-learning-based distinction (Naïve Bayes) was used to identify AD^3,29^.

A multilayer perceptron (MLP) is a class of feedforward artificial neural network that has been used in diverse fields, and it can be utilized to construct effective classifier algorithms for distinguishing data that are not linearly separable. The aim of the present study was to determine the effectiveness of using arterial pulse-wave measurements in discriminating between AD patients and control subjects. Arterial pulse waveforms were acquired noninvasively in mechanical measurements (using strain-gauge sensors to measure the BPW) and optical measurements (in PPG). The analyses focused on frequency-domain changes in the BPW and PPG pulse waveforms. An MLP algorithm was used with the aim of providing a classification for AD. The obtained knowledge may be pertinent to the development of a noninvasive and easy-to-use measurement technique for detecting changes induced by AD on the arterial pulse transmission condition.

## Methods

Details of the present experimental setup and the signal processing methods are available elsewhere^17,19,22^. This study investigated the potential of using pulse indices to specifically identify AD patients in a sample of 87 AD patients and 74 control subjects. Based on Mini Mental State Examination (MMSE) scores, the patients (whose details are listed in Table 1) were divided into three subgroups: mild AD (Group I; MMSE scores > 16 and ≤ 24), moderate AD (Group II; MMSE scores > 10 and ≤ 16), and severe AD (Group III; MMSE scores ≤ 10).

**Table 1:** Characteristics of subjects.

	AD						Control	
	16 < MMSE < 24 (mild AD)		10 < MMSE ≤ 16 (moderate AD)		MMSE ≤ 10 (severe AD)			
Gender	Male	Female	Male	Female	Male	Female	Male	Female
Subject number	14	18	23	18	7	7	22	52
	32		41		14		74	
	87							
Average age	69.86 ± 8.65	74.83 ± 6.60	77.39 ± 9.77	77.61 ± 8.74	76.28 ± 5.36	85.14 ± 3.98	70.06 ± 3.14	70.07 ± 3.74
	72.65 ± 7.96		77.48 ± 9.34		80.71 ± 6.47		70.07 ± 3.58	
	76.20 ± 8.88							
Average heart rate	63.85 ± 8.24	68.79 ± 5.08	66.35 ± 12.32	68.32 ± 10.16	62.88 ± 5.82	65.06 ± 5.01	72.73 ± 11.91	77.13 ± 9.99
	66.48 ± 7.71		67.25 ± 12.45		63.97 ± 5.54		75.82 ± 10.79	
	66.40 ± 9.24							
BMI	23.54 ± 1.97	23.38 ± 3.58	21.46 ± 2.22	22.45 ± 2.96	22.18 ± 1.41	18.86 ± 0.82	26.12 ± 2.88	25.52 ± 4.24
	23.45 ± 2.96		21.94 ± 2.66		21.07 ± 2.00		25.70 ± 3.89	
	22.38 ± 2.85							
Hypertension	7	10	9	10	2	2		
	17		19		4			
	40							
Hyperlipidemia	5	13	8	8	3	2		
	18		16		5			
	39							

The subjects were recruited in the Ren-Ai Branch of Taipei City Hospital. Written informed consent was obtained from the study participants or their legal designates to publish the information in an online open access publication (approved by the Review Board of Taipei City Hospital; TCHIRB-10810016-E). All experiments were performed in accordance with relevant guidelines and regulations. The inclusion criteria were age ≥ 50 years and diagnosed as AD by the neurologist or psychiatrist. Psychologists evaluated the severity and disability of patients with dementia, and those with an MMSE score of < 25 or a Clinical Dementia Rating of > 0.5 were eligible to participate in the study. Patients were excluded if they did not agree to participate in the study or were unable to cooperate with the research steps, such as due to their limbs trembling involuntarily, restlessness, or agitated movement.

### Measurements

**Figure 1:**
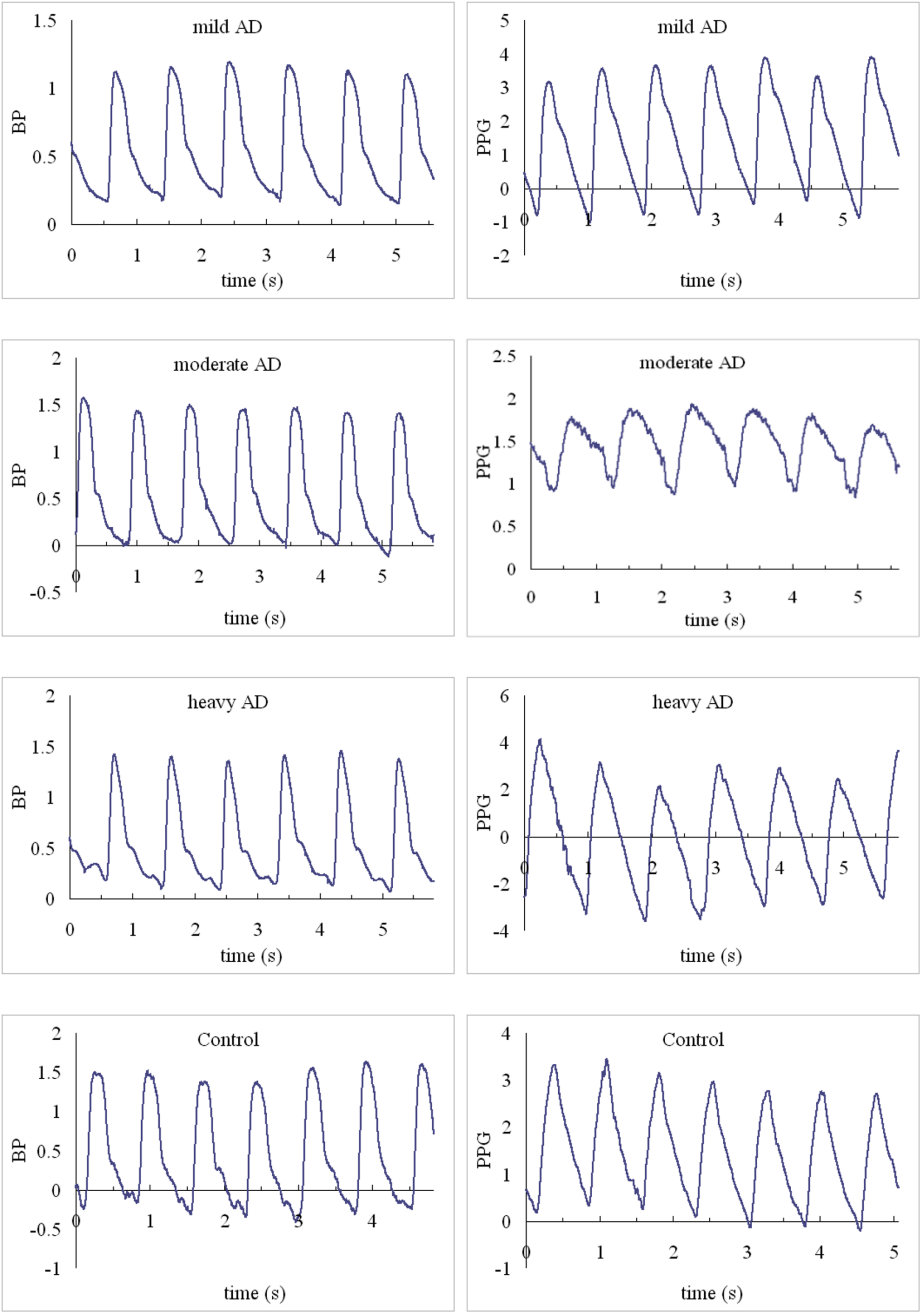
Typical measured pulse waveforms.

BPW and PPG signals were noninvasively measured in the subjects (typical waveforms were shown in Fig. 1). Before the measurements, the subjects were relaxed and rested for 20 min. For each experiment, the subjects were sitting on a chair, and we recorded a 3-min data sequence. The BPW signal was acquired by a pressure transducer (KFG-2-120-D1-11, Kyowa) held onto the skin surface above the radial artery 2 cm from the left wrist^20^. The PPG signal from a 940-nm-wavelength infrared LED penetrating the right middle finger tissue was acquired by a photodiode^20^. These signals were sampled at 1024 Hz. Before the measurement, the heart rate (HR), brachial systolic BP and diastolic BP were measured by using a sphygmomanometer (MG150f, Rossmax).

### Analysis

There are two parts in the present analysis procedure: signal processing and information processing.

#### Signal processing

When determining the pulse waveforms, the signals were filtered by a digital 11th-order high-pass Chebyshev filter with a cut-off frequency of 0.01 Hz to eliminate the baseline drift and to provide a steeper transition region to improve the filtering effects on lower-frequency interference (such as motion artifact)^17,20^. Frequency-domain analysis was applied to derive the following four harmonic indices from the measured BPW and PPG signals: amplitude proportion (*C*_*n*_), coefficient of variation of *C*_*n*_ (*CV*_*n*_), phase angle (*P*_*n*_), and standard deviation of *P*_*n*_ (*P*_*n*__*SD*).

Each individual pulse (between foot points) can be represented by the following finite series^20^:

$$ x(t) = \frac{{A_{0} }}{2} + \left\{ {\sum\limits_{n = 1}^{k/2} {A_{n} \cos n \, \omega \, t_{s} + } \sum\limits_{n = 1}^{k/2} {B_{n} \sin n \, wt_{s} } } \right\}. $$

The Fourier coefficients (*A*_*n*_ and *B*_*n*_) of the pulse can be calculated as

$$ \begin{aligned} & A_{n} = \frac{2}{k}\sum\limits_{s = 0}^{k} {x_{s} \cos \,n \, \omega \, t_{s} } \, \left( { \, for\, \, n = 0,1,...,\frac{k}{2}} \right) \\ & B_{n} = \frac{2}{k}\sum\limits_{s = 0}^{k} {x_{s} \sin \,n \, \omega \, t_{s} } \, \left( { \, for \, \,n = 0,1,...,\frac{k}{2} \, } \right), \\ \end{aligned} $$

where $\omega$ is the angular frequency and $t_{s}$ is the sampling time interval.

The amplitude (*Amp*_*n*_; *n*: the harmonic number) and phase angle (*P*_*n*_) of each harmonic of the pulse harmonic spectrum can then be calculated as $Amp_{n} = \sqrt {A_{n}^{2} + B_{n}^{2} }$ and $P_{n} = \arctan (B_{n} /A_{n} )$. The amplitude proportions (*C*_*n*_ values) for each pulse were calculated as *Amp*_*n*_/*Amp*_0_ × 100%, for *n* = 1–10. *CV*_n_ was then calculated as the coefficient of variations (CV) of *C*_*n*_, and *P*_n__*SD* was calculated as the standard deviation (SD) of *P*_n_.

Signal processing was performed with MATLAB (MathWorks). The differences were tested with two-tailed t-test and were considered significant when *p* < 0.05; all *p*-values were two-sided hypotheses.

#### Information processing

For information processing, the features of pulse signals were collected from the results of the signal-processing stage described above, to yield 40 indices for each pulse: *C*_*n*_, *CV*_*n*_, *P*_*n*_, and *P*_*n*__*SD* values for *n* = 1–10. Before being input to the MLP analysis as features, *CV*_*n*_ and *P*_*n*__*SD* were recalculated for each pulse and the following 14 pulses. Each feature was scaled by *Z*-score normalization to eliminate the effects of the variations in the ranges of different indices.

Python (version 3.7) was used as the analysis tool in the information processing, and an MLP with supervised learning was used to classify the data. In addition to an input layer (40 nodes) and an output layer, there were 3 hidden layers (20 nodes for each layer). The AD patients and control subjects were randomly split into training and validation sets at a ratio of 2:1. Since 3-min recordings were available for each subject, there were around 180–250 pulses in the data of each subject.

Threefold cross-validation was used in the model training process, with 80% and 20% of subjects in the training set assigned to training and validation, respectively. Hold-out analysis was performed to evaluate the model accuracy (analysis procedure was shown in the Supplemental Information). Training and validation accuracy plots were used to monitor the learning dynamics. The classification ability of the proposed classification model was evaluated by calculating the accuracy, sensitivity, specificity, and AUC (area under the receiver operating characteristics curve), and the confusion matrix was determined to evaluate the classifier performance.

## Results and discussion

The present study found significant differences in several BPW and PPG indices between AD patients and control subjects. The MLP analysis indicated that these indices can be used to aid the identification of AD.

### AD vs control subjects

**Figure 2:**
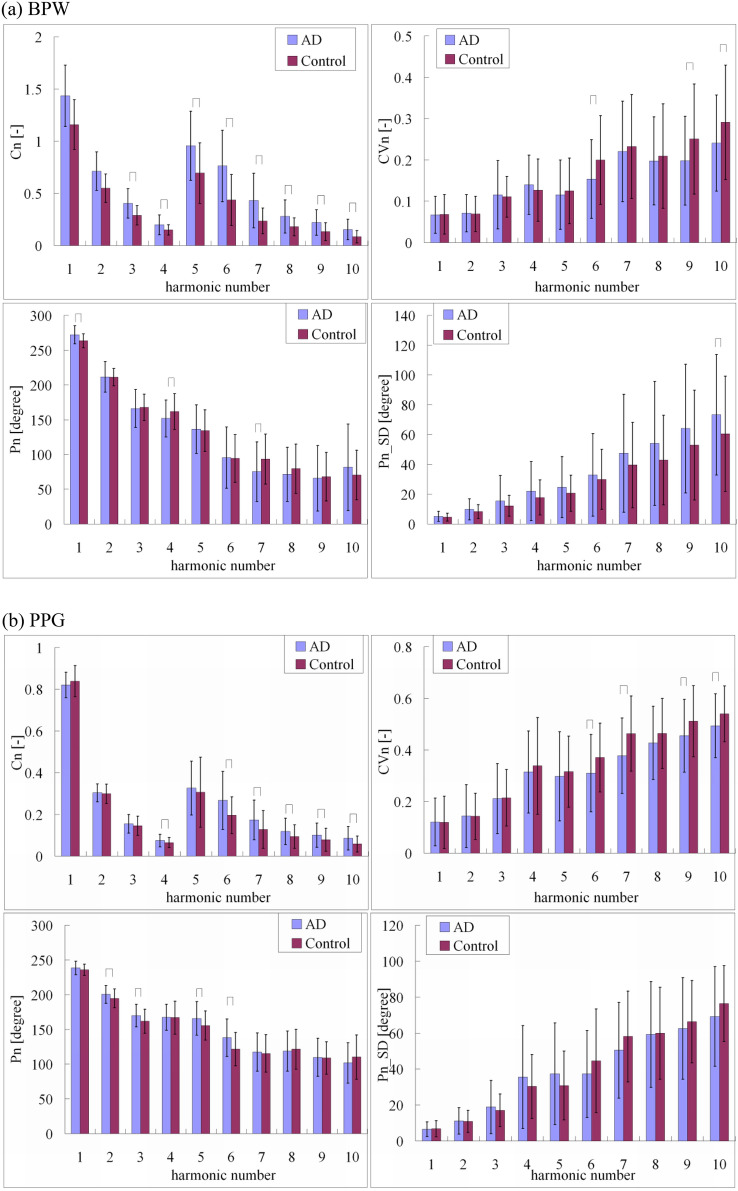
Comparisons of (**a**) BPW and (**b**) PPG harmonic indices between AD patients and control subjects: *C*_*n*_, *CV*_*n*_, *P*_*n*_, and *P*_*n*__*SD*. Data are mean and standard-deviation values. *C*_5_–*C*_10_ values have been multiplied by 5 to make the differences clearer. “⌢” indicates *p* < 0.05. In BPW indices, *C*_3_–*C*_10_, *P*_1_, and *P*_10__*SD* were significantly larger and *CV*_6_, *CV*_9_, *CV*_10_, *P*_4_, and *P*_7_ were significantly smaller in AD patients than the control subjects. In PPG indices, *C*_4_, *C*_6_–*C*_10_, *P*_2_, *P*_3_, *P*_5_, and *P*_6_ were significantly larger and *CV*_6_, *CV*_7_, *CV*_9_, and *CV*_10_ were significantly smaller in AD patients than the control subjects.

The characteristics of the study subjects are listed in Table 1. Figure 2a compares the harmonic indices of the BPW signals. Most of the amplitude ratios (*C*_3_–*C*_10_) were significantly larger in the AD patients (for example, *C*_3_: 39.22%) than the control subjects. *P*_1_ (8.67 degrees) and *P*_10__*SD* (21.20%) were significantly larger while *CV*_6_ (23.13%), *CV*_9_ (20.99%), *CV*_10_ (17.24%), *P*_4_ (10.04 degrees), and *P*_7_ (17.99 degrees) were significantly smaller in the AD patients than the control subjects.

Cerebrovascular atherosclerosis has been identified as a prominent pathological feature of AD, with large- and small-cerebral-vessel pathology being associated with worse cognitive performance and an increased risk for AD, and this link may also extend to extracranial arteries^2^. Since the noise present in the time-domain pulse waveform may interfere with the determination of foot or peak points, which are important in determining the time-domain indices such as the pulse wave velocity and augmentation index, frequency-domain analysis might be useful for evaluating the vascular stiffness. The present study has further revealed a noninvasive method that involves measuring and analyzing the pulse waveform that may helpful for monitoring AD-induced changes in the vascular stiffness.

The above conjecture provides a possible explanation for the present findings in the pulse indices acquired at around the wrist and the finger. Figure 2a shows that the *C*_*n*_ values in BPW for seven frequency components were significantly larger in AD patients than the control subjects, which reflects changes in the transmission condition of the arterial pulse wave. The increased arterial stiffness accompanying AD may increase the transmission efficiency of the arterial pulse so as to increase the amplitude ratios of harmonic components.

Phase angle *P*_*n*_ is related to the rise time of each frequency component in the pulse waveform, with a larger *P*_*n*_ corresponding to an earlier increase in the *n*th harmonic component^20^. A stiffened artery may increase the transmission speed of the main (i.e., first) frequency component, hence leading to the increase in the value of *P*_1_.

Variability indices such as heart-rate and BP variability^30,31^ have been used in many studies to aid the monitoring of the cardiovascular regulatory activities. *P*_*n*__*SD* is defined as the variability of *P*_*n*_. Since the phase angle is related to the rise time and thus the transmission speed of frequency components in the pulse waveform, *P*_*n*__*SD* may be useful for monitoring the cardiovascular regulatory activities that affect the vascular elastic properties.

Figure 2a shows that *P*_*n*__*SD* was larger for all harmonic components in AD patients than the control subjects (although the difference was only significant for *P*_10__*SD*). This illustrates the presence of larger regulatory activities acting on the vascular elastic properties. It is possible that when facing an induced increase in vascular stiffness, more effort is needed from the vascular regulation mechanism to deal with the changes in the blood-flow perfusion condition. This situation might increase the time variation of the vascular stiffness, and thus increase the *P*_*n*__*SD* values.

The significant differences found in various BPW pulse indices between AD patients and control subjects prompted us to perform further MLP analyses in an attempt to provide an effective method for discriminating these two groups. Table 2 shows the MLP analysis results for the comparison of BPW indices between the AD patients and control subjects. For the threefold cross-validation, the mean accuracy, sensitivity, specificity, and AUC were 89.83%, 81.00%, 98.67%, and 0.90, respectively.

**Table 2:** MLP analysis results for comparisons of BPW and PPG indices between AD patients and control subjects.

	1	2	3	Average
**BPW**				
Accuracy (%)	90.00	92.00	87.50	89.83
Sensitivity (%)	80.00	88.00	75.00	81.00
Specificity (%)	100.00	96.00	100.00	98.67
AUC	0.90	0.92	0.88	0.90
**PPG**				
Accuracy (%)	70.83	63.83	60.98	65.21
Sensitivity (%)	72.00	50.00	70.00	64.00
Specificity (%)	77.67	78.26	52.38	69.44
AUC	0.71	0.64	0.61	0.65

Training and validation results are presented for the threefold cross-validation. For BPW indices, there were 6247 and 6626 pulses for AD patients and control subjects, respectively. For PPG indices, there were 4673 and 4043 pulses for AD patients and control subjects, respectively.

For the BPW indices, we performed hold-out analysis to further evaluate the model accuracy. As listed in Table 3, the mean accuracy, sensitivity, specificity, and AUC were 74.23%, 72.140%, 79.40%, and 0.74, respectively, for the threefold cross validation. The mean accuracy, sensitivity, specificity, and AUC were 82.86%, 77.72%, 92.31%, and 0.83, respectively, for the hold-out test. These values indicate that when using BPW pulse indices as features, MLP analysis facilitates the ability to distinguish AD patients from control subjects with high accuracy and a very high specificity.

**Table 3:** Hold-out MLP analysis results for comparisons of BPW indices between AD patients and control subjects.

BPW	Threefold cross validation				Hold-out test
	1	2	3	Average	
Accuracy (%)	76.92	76.32	69.44	74.23	82.86
Sensitivity (%)	70.83	75	70.59	72.14	77.72
Specificity (%)	92.00	77.78	68.42	79.40	92.31
AUC	0.77	0.76	0.69	0.74	0.83

As illustrated in Fig. 2b, the PPG indices showed similar changing trends as the BPW indices, although the differences were less prominent. Only *C*_4_ and *C*_6_–*C*_10_ (for example, *C*_4_: 15.59%) were significantly larger in the AD patients than the control subjects. *P*_2_ (5.85 degrees), *P*_3_ (8.11 degrees), *P*_5_ (10.18 degrees), and *P*_6_ (16.50 degrees) were significantly larger while *CV*_6_ (16.22%), *CV*_7_ (18.53%), *CV*_9_ (11.00%), and *CV*_10_ (8.52%) were significantly smaller in AD patients than the control subjects. The PPG measurement site (at the finger) is more distal (from the heart) than the BPW measurement site, which means that the measurement site is further from the cerebrovascular beds. Moreover, the BPW measurement site is close to the main artery, which decreases the effects induced by AD on the pulse waveform. These factors may together partly account for the effects of AD appearing less prominently (although being similar) in the PPG pulse indices than the BPW pulse indices.

Table 2 also illustrates that the differences in the PPG indices between the AD patients and control subjects were less prominent than those in the BPW indices. There were also similar results in the MLP analysis: for the threefold cross-validation results as shown in Table 2, the mean accuracy, sensitivity, specificity, and AUC were 65.21%, 64.00%, 69.44%, and 0.65, respectively. These values indicate that the discrimination accuracy of the PPG indices was inferior to that of the BPW indices. Therefore, using the PPG indices might be less effective than using the BPW pulse indices as features to perform MLP-based classification for distinguishing between AD patients and control subjects.

### Comparison between mild, moderate, and severe AD

A link between aortic stiffness and worse cognitive function was found in the Rotterdam Study^32^. It has been suggested that vascular pathology can amplify the deleterious effects of AD pathology^1^. Pathology of large cerebral vessels (atherosclerosis) and small cerebral vessels (arteriolosclerosis) is associated with worse cognitive performance and an increased risk of AD^33^. The underlying mechanisms may involve impaired Aβ clearance resulting in inflammation not being resolved^34^. Hypoperfusion and hypoxia enhance Aβ production in severe atherosclerosis, which may in turn promote the formation of atherosclerotic lesions^34^.

**Figure 3:**
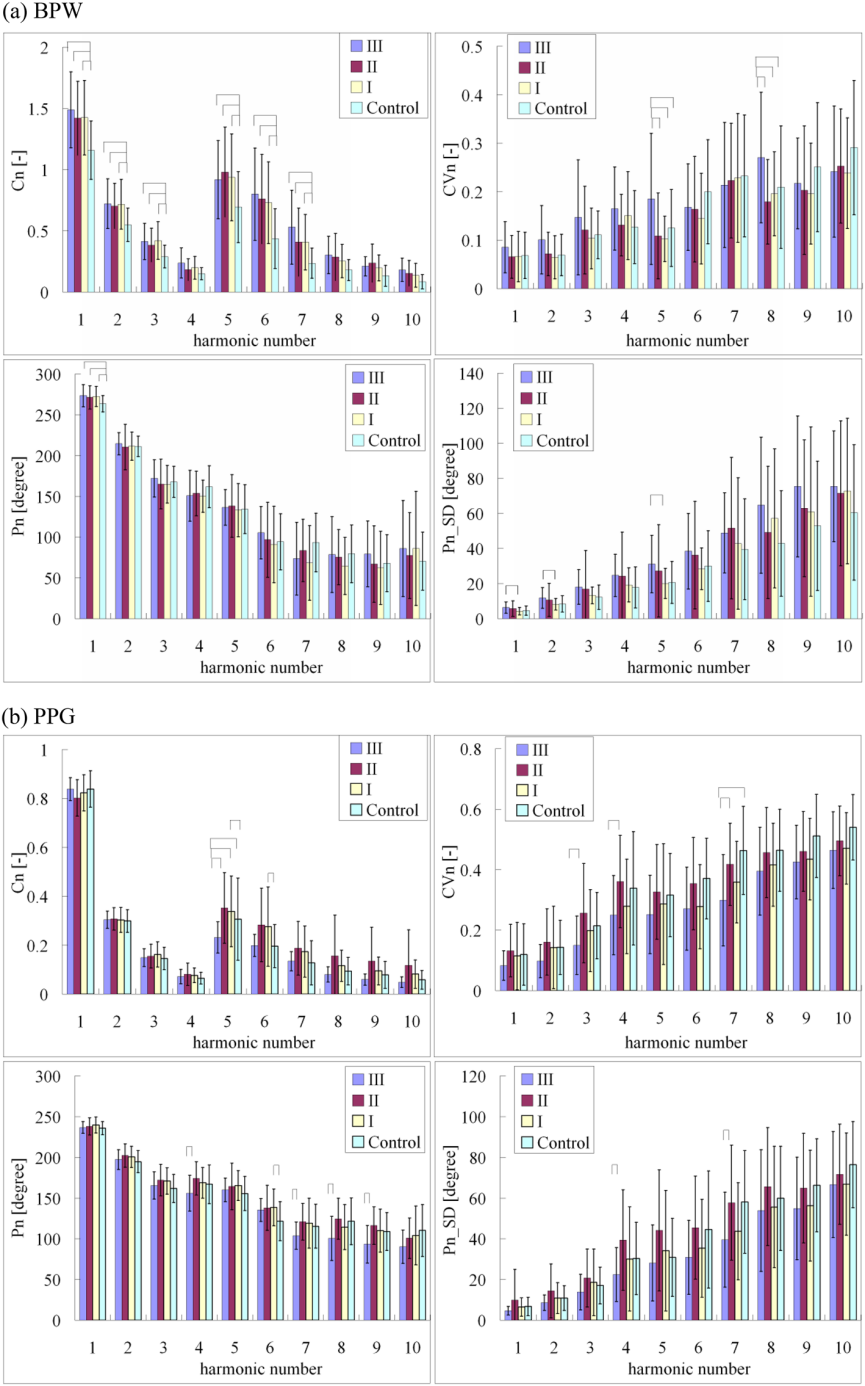
Comparisons of (**a**) BPW and (**b**) PPG harmonic indices between patients with severe AD (Group III), moderate AD (Group II), and mild AD (Group I), and control subjects: *C*_*n*_, *CV*_*n*_, *P*_*n*_, and *P*_*n*__*SD*. Data are mean and standard-deviation values. *C*_5_–*C*_10_ values have been multiplied by 5 to make the differences clearer. “⌢” indicates *p* < 0.05. For BPW indices, *C*_1_–*C*_3_, *C*_5_–*C*_10_, and *P*_1_ were significantly larger in Group III than the control subjects. *CV*_5_ and *CV*_8_ were significantly larger in Group III than in Group II, Group I, and the control subjects. For PPG indices, there were significant differences between Groups II (moderate AD) and III (severe AD) in *C*_5_, *CV*_3_, *CV*_4_, *CV*_7_, *P*_4_, *P*_7_, *P*_8_, *P*_9_, *P*_4__*SD*, and *P*_7__*SD*.

Figure 3a compares the harmonic indices of the BPW signals between patients with severe, moderate, and mild AD, and control subjects. The amplitude ratios for all harmonic components except for *C*_4_ were significantly larger for the three AD subgroups than for the control subjects. The phase angle of the main frequency component (*P*_1_) of the subgroups was also significantly larger in AD patients than the control subjects. These trends were similar to those in Fig. 2a, which indicates the similarity of the effects induced by AD on arterial pulse transmission and the BPW waveform.

For the variability indices, there were larger variability values in some frequency components in AD Group I than in Group III. For example, *CV*_5_ (48.12%), *CV*_8_ (29.01%), *P*_1__*SD* (37.04%), *P*_2__*SD* (39.91%), and *P*_5__*SD* (50.77%) were significantly larger in Group I. This suggests that in addition to discriminating between AD patients and control subjects, the present method of pulse waveform analysis can be used to distinguish between severe AD and mild AD. We suggest that further MLP analyses should be performed to verify this possibility.

Table 4 shows the MLP analysis results for the comparison of BPW indices between mild and severe AD. For the threefold cross-validation, the mean accuracy, sensitivity, specificity, and AUC were 68.09%, 65.00%, 70.00%, and 0.68, respectively. These values indicate that the BPW indices can be used to discriminate between severe AD and mild AD. The discrimination accuracy of the BPW analysis was not as good as that between AD patients and control subjects, which could be partly attributed to the smaller sample of the three AD subgroups compared to including all of the AD patients. Further efforts could be focused on accumulating more AD patients to verify the present conjecture.

**Table 4:** MLP analysis results for comparisons of BPW and PPG indices between patients with mild and severe AD.

	1	2	3	Average
**BPW**				
Accuracy (%)	66.67	64.30	73.30	68.09
Sensitivity (%)	60.00	75.00	60.00	65.00
Specificity (%)	70.00	60.00	803.00	70.00
AUC	0.65	0.68	0.70	0.68
**PPG**				
Accuracy (%)	80.00	71.43	71.43	74.28
Sensitivity (%)	40.00	60.00	20.00	40.00
Specificity (%)	100.00	77.78	100.00	92.59
AUC	0.70	0.69	0.60	0.66

Training and validation results are presented for the threefold cross-validation. For BPW indices, there were 941 and 913 pulses for severe and mild AD, respectively. For PPG indices, there were 637 and 634 pulses for severe and moderate AD, respectively.

Figure 3b compares the harmonic indices of the PPG signals between patients with severe, moderate, and mild AD, and control subjects. The differences in the amplitude ratios were not as prominent as for the BPW indices, but more of the indices differed significantly between severe and moderate AD in several PPG indices: *C*_5_ (24.36%), *CV*_3_ (30.29%), *CV*_4_ (26.30%), *CV*_7_ (35.29%), *P*_4_ (11.00 degrees), *P*_7_ (11.62 degrees), *P*_8_ (21.08 degrees), *P*_9_ (15.69 degrees), *P*_4__*SD* (25.95%), and *P*_7__*SD* (32.02%). These findings imply that the present PPG indices may be useful for discriminating between moderate and severe AD, and we plan to perform further MLP analyses to verify this possibility.

Table 4 also shows the MLP analysis results for the comparison of PPG indices between moderate and severe AD. For the threefold cross-validation, the mean accuracy, sensitivity, specificity, and AUC were 74.28%, 40.00%, 92.59%, and 0.66, respectively. These findings indicate that the PPG indices can be used to facilitate the discrimination between severe and moderate AD with a very high specificity.

**Figure 4:**
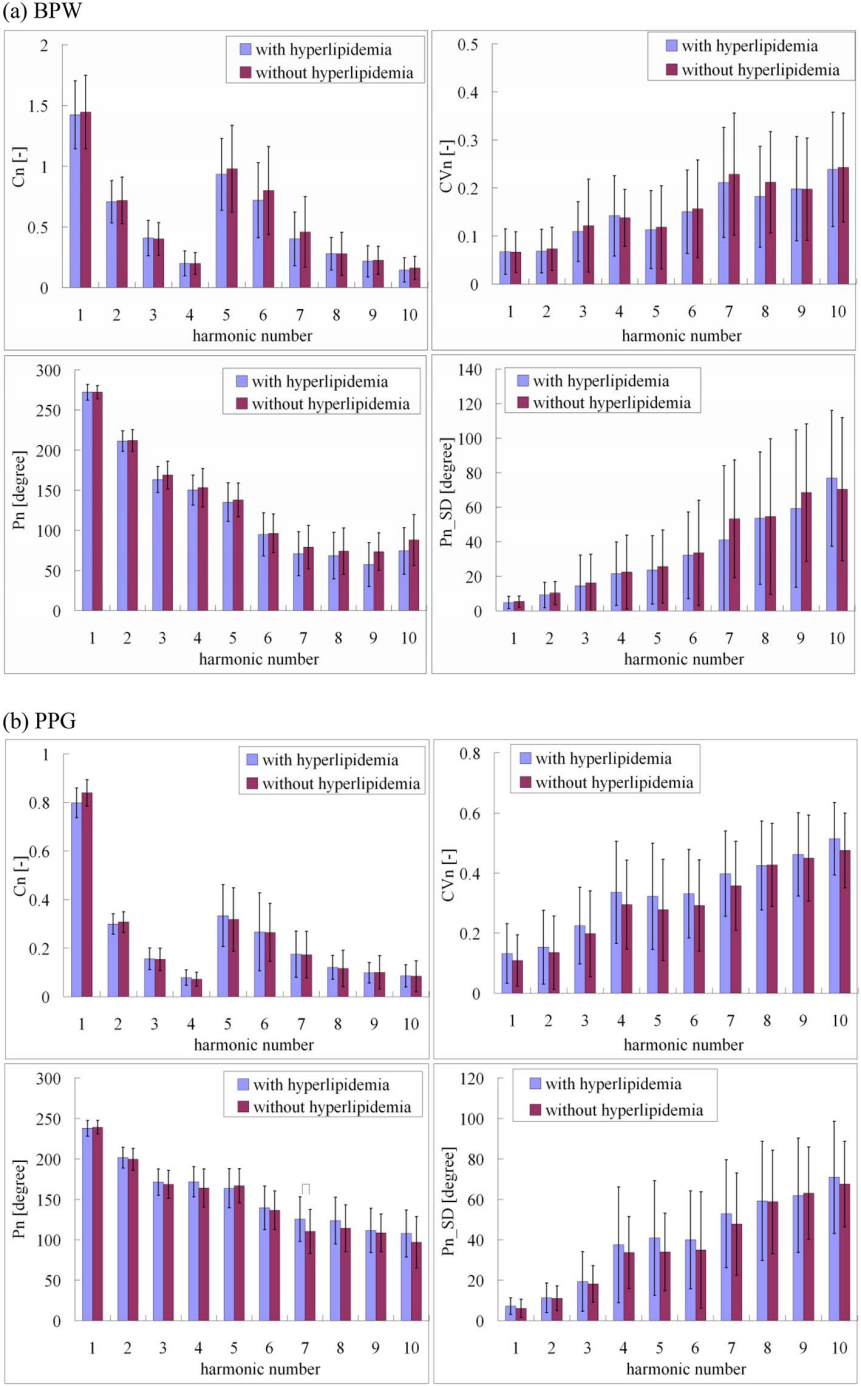
Comparisons of (**a**) BPW and (**b**) PPG harmonic indices between subjects with and without hyperlipidemia: *C*_*n*_, *CV*_*n*_, *P*_*n*_, and *P*_*n*__*SD*. Data are mean and standard-deviation values. *C*_5_–*C*_10_ values have been multiplied by 5 to make the differences clearer. “⌢” indicates *p* < 0.05. None of the BPW pulse indices differed significantly between subjects with and without hyperlipidemia. *P*_7_ was the only PPG pulse index that differed significantly between subjects with and without hyperlipidemia.

Figure 4 and Table 4 together illustrate that while the number of subjects was relatively small, combining BPW and PPG measurements and analyses may provide an effective discrimination method for different disease-progression states of AD: severe and mild AD using BPW indices, and severe and moderate AD using PPG indices. As described above, the underlying mechanism may be partly attributed to the effects induced by AD on the vascular elastic properties differing between the AD subgroups with different levels of disease progression^2^.

Many studies have proposed AD diagnostic applications in collaboration with machine-learning methods; machine-learning have been to diverse data categories of neuroimaging, cognitive assessment data and speech analysis of AD^26^. Initial screening of dementia patients is generally performed through the use of various neuropsychological assessments; a review of clinical trials evaluating the MMSE produces results ranging from 21 to 100% in sensitivity and 46–100% in specificity^35^. Improvements in classification accuracy were reported amongst several machine-learning methods ranging from 63 to 76%; SVM and NN show high sensitivities nearing 100% but fall short in sensitivity dropping below 50%^36^. So et al. performed a two stage evaluation process: in Stage-1 (consisted of 9799 control subjects and 4201 MCI patients) results, NNs can achieve an accuracy of 97.2% (sensitivity: 97%; specificity: 96%); in Stage-2 (consists of 663 MCI and 573 dementia subjects) results, SVM can achieve an accuracy of 74.0% (sensitivity: 73.8%; specificity: 73.8%)^37^. Zhu et al. developed a method based on machine learning to help the preliminary diagnosis of normal, MCI, VMD, and dementia using an informant-based questionnaire; the Naive Bayes algorithm can achieve an 81% accuracy (precision: 0.82, recall: 0.81, and F-measure: 0.81)^27^.

AD diagnosis using machine learning with MRI scans has been proven to be extremely effective in diagnostic ability. For example, Kloppel et al. compared the diagnostic performance between a SVM model and six expert radiologists in 52 confirmed AD cases with an equal, age matched control and fronto-temporal lobar degeneration samples. The SVM model can achieve an accuracy of 92.4% (sensitivity: 92.8%, specificity: 91.8%), which was able to outperform human radiologists with an 80.0% accuracy (sensitivity: 77.2%, specificity: 82.0%)^28^. Semantic dementia is characterized by cognitive deterioration involving semantic memory, language and perceptual processes, which provide a possible way to facilitate noninvasive diagnosis technique^26^. Jarrold et al. attempted a combination of acoustic-level and lexical feature extraction; NN was reported to have an 88% accuracy in discrimination between AD and control^38^.

Compared with the results in these previous studies, the present MLP-based method can have similar performance in AD discrimination. Therefore the present measurement and analysis technique can help to provide another noninvasive, rapid, and objective method to aid the detection or screening of AD status. The sample size was a major drawback for the present comparisons between AD subgroups. Future efforts could therefore focus on accumulating more AD patients in order to increase the numbers of subjects in the subgroups with different disease-progression states in order to verify the present conjecture.

### Effects of possible interfering factors

There are many factors that may affect the vascular stiffness, including hyperlipidemia^39^, age^40^, and hypertension^40,41^. These factors may change the transmission condition for the arterial pulse, and thus further change the pulse waveform and its indices. This may impair the identification accuracy of the method of MLP analysis based on using pulse indices as features. It is therefore necessary to examine the possible effects of these factors on the pulse indices.

Most of the available data provides evidence of a positive relationship between plasma cholesterol and arterial stiffness, and also a link between plasma cholesterol and BP^39^. Figure 4 divide the AD patients into those with and without hyperlipidemia, and compare the harmonic indices of the BPW and PPG signals between them. There were no significant differences in any of the pulse indices except for *P*_7_ (15.34 degrees) in PPG. This indicates that there was no prominent effect induced by the hyperlipidemia factor, which implied that this did not interfere markedly with the MLP-based classification ability for the AD patients.

Recent studies have shown that arterial stiffening precedes the development of a high BP, and can be used to predict future cardiovascular events^40^. Arterial stiffness has been recognized as an important physiopathological determinant for the age-related increase in systolic BP^41^. An increased BP may distend vessels, and hypertension is associated with vascular dysfunction, with changes at both the histological and cellular levels in vessels^42^. These observations imply that hypertension may change the arterial stiffness and thus the values of pulse indices in the AD patients.

**Figure 5:**
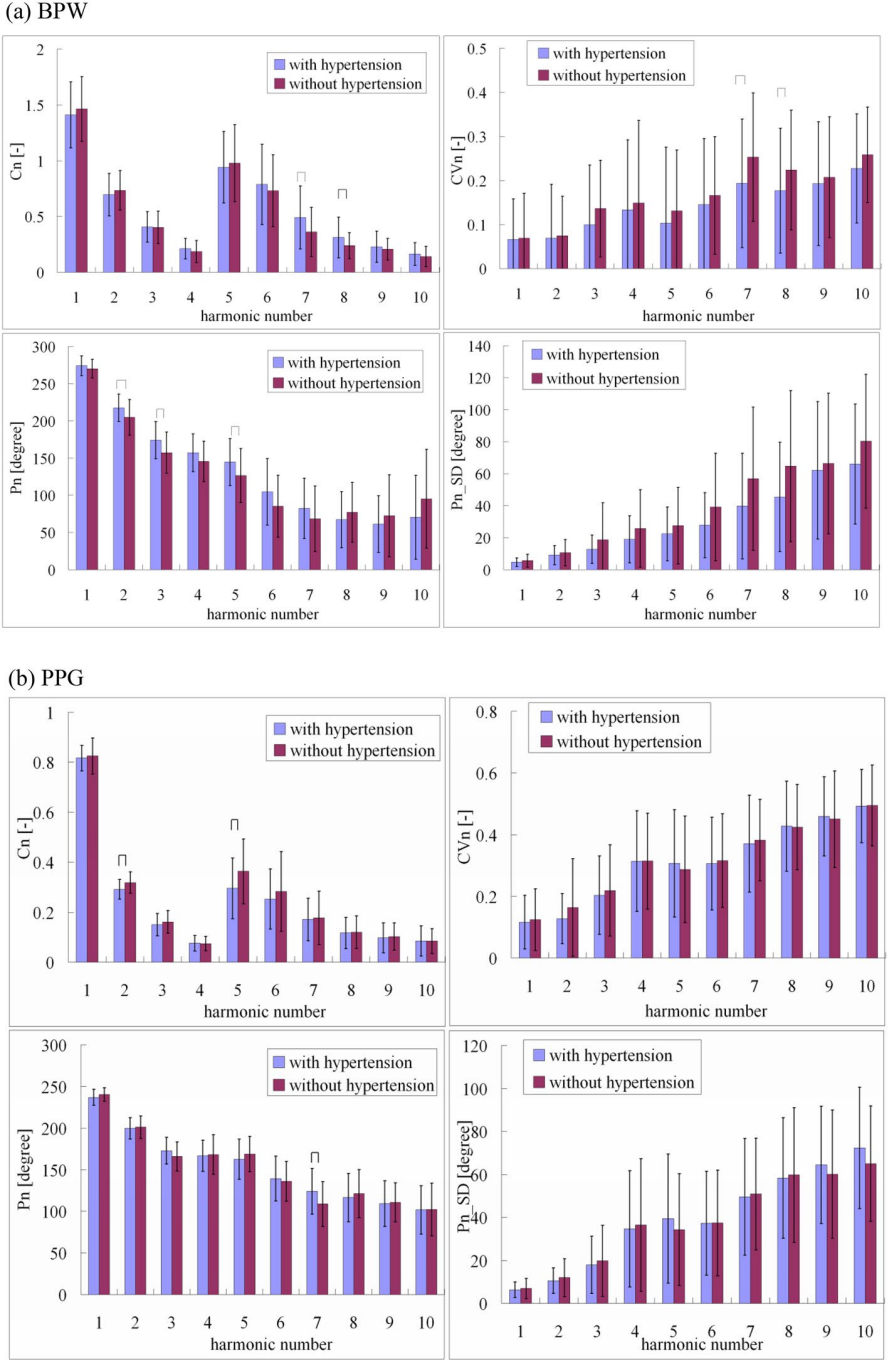
Comparisons of (**a**) BPW and (**b**) PPG harmonic indices between subjects with and without hypertension: *C*_*n*_, *CV*_*n*_, *P*_*n*_, and *P*_*n*__*SD*. Data are mean and standard-deviation values. *C*_5_–*C*_10_ values have been multiplied by 5 to make the differences clearer. “⌢” indicates *p* < 0.05. In BPW indices, only *C*_7_, *C*_8_, *CV*_7_, *CV*_8_, *P*_2_, *P*_3_, and *P*_5_ differed significantly between subjects with and without hypertension. In PPG indices, only *C*_2_, *C*_5_, and *P*_7_ differed significantly between subjects with and without hypertension.

Figure 5 compare the harmonic indices of the BPW and PPG signals between subjects with and without hypertension. Similar to the effects of hyperlipidemia, significant differences were revealed for only a few of the pulse indices between subjects with and without hypertension. This again illustrates that no marked effect was induced by hypertension, which implied no prominent interference with the MLP-based discriminating ability for the AD patients.

The arterial stiffness is reported to be positively associated with aging^22^. Subjects of different ages may therefore exhibit different levels of vascular stiffness, which could interfere with the AD discriminating ability of using pulse indices as features. To evaluate these possible age-related effects, we compared the mean ages between the correctly classified and incorrectly classified AD patients. When using the BPW indices, the correctly classified AD patients were aged 76.03 ± 8.91 years (mean ± standard deviation) while the incorrectly classified AD patients were 77.38 ± 5.86 years old (*p* = 0.607 by two-tailed *t*-test). For the PPG indices, the ages of the correctly and incorrectly classified AD patients were 74.63 ± 7.67 and 77.85 ± 9.60 years, respectively (*p* = 0.136 by two-tailed *t*-test). The absence of significant differences in the ages between correctly and incorrectly classified AD patients indicates that the subject age did not markedly interfere with the MLP-based classification for AD when using pulse indices as features (Supplementary Information).

## Conclusion

The findings of this study and the related conclusions to be drawn can be summarized as follows:

1. Significant differences in BPW and PPG pulse indices were found between the AD patients and control subjects. These differences can be partly attributed to AD-induced changes in the vascular elastic properties.
2. The hold-out results indicated that the present method of MLP analysis using spectral BPW pulse indices as features is highly effective at distinguishing between AD patients and control subjects, with an accuracy of over 80% and a high specificity (> 90%).
3. The present method of MLP analysis using BPW and PPG indices as features can distinguish between mild, moderate, and severe AD with an accuracy of around 70%. Future efforts can be focused on accumulating more AD patients in order to increase the numbers of subjects in the subgroups with different disease-progression states in order to verify the present conjecture.
4. In the present results, age, hypertension, and hyperlipidemia did not markedly interfere with the pulse indices. Further collection of more AD-patient data is necessary to understand the possible interference effects of these factors.
5. The present findings may be meaningful for the development of a noninvasive, rapid, inexpensive, and objective method based on MLP analysis to detect and monitor the AD status.

## Supplementary Information

Supplementary Information.

## Data Availability

The datasets generated during and/or analyzed during the current study are available from the corresponding author on reasonable request.
