# Supplementary material for: Classification of patients with Alzheimer’s disease using the arterial pulse spectrum and a multilayer-perceptron analysis
Source: Sci Rep. 2021 Apr 26;11:8882. doi: 10.1038/s41598-021-87903-7 (PMC8076260; doi:10.1038/s41598-021-87903-7)

Title: Classification of patients with Alzheimer’s disease using the arterial pulse spectrum and a multilayer-perceptron analysis

Short title: MLP-based classification of the pulse spectrum in AD

Authors: Shun-Ku Lin^1-3^, Hsin Hsiu^4, 5^, Hsi-Sheng Chen^4^, Chang-Jen Yang^4^

1. Institute of Public Health, National Yang-Ming University, Taipei, Taiwan.

2. Department of Chinese medicine, Taipei City Hospital, Renai Branch, Taipei, Taiwan.

3. General Education Center, University of Taipei, Taipei, Taiwan.

4. Graduate Institute of Biomedical Engineering, National Taiwan University of Science and Technology, Taipei, Taiwan.

5. Biomedical Engineering Research Center, National Defense Medical Center, Taipei, Taiwan.

To whom correspondence should be addressed: Hsin Hsiu, PhD.

No.43, Section 4, Keelung Road, Graduate Institute of Biomedical Engineering, National Taiwan University of Science and Technology, Taipei 10607, Taiwan.

Tel: +1 886 22730- 3730; Fax: +1 886 2 2730-3733; E-mail: hhsiu@mail.ntust.edu.tw

Flowchart for (a) Measurement. (b) Signal processing: 40 indices (*C_n_*, *P_n_*, *CV*_n_, and *P*n_*SD*) were calculated; these indices were used as the features for the information processing. (c) Information processing: threefold cross validation to evaluate the model performance and then hold-out analysis to test model accuracy.

Scikit-learn preprocessing package was used in the normalization. StandardScaler() was used to standardize features; fit.transform() and transform() were used to scale the training and test data. Between layers, batch normalization was performed to accelerate learning and to reduce the over-fitting. Relu activation function, dropout() (dropout rate=0.3) and kernel_initializer were used.

In the output layer, we used sigmoid activation function for binary classification. model.evaluate() was used to evaluate the model on the test data. Adam optimizer was used; the cross entropy was used as the loss function. Batch size=500; epochs=300.

In hold-out analysis, 1/4 of the subjects were randomly chosen as the hold-out set. Threefold cross validation was performed on the data of the remaining 3/4 subjects to build up the model. Then the hold-out set was used to test the model accuracy.

(a)


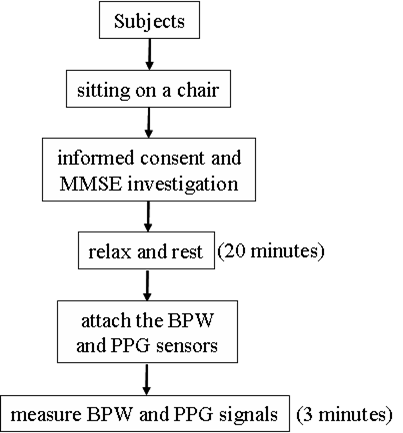


(b)


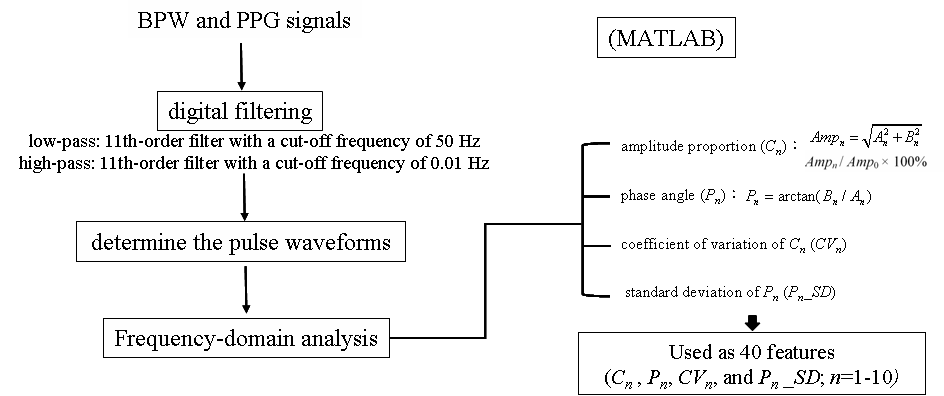


(c)


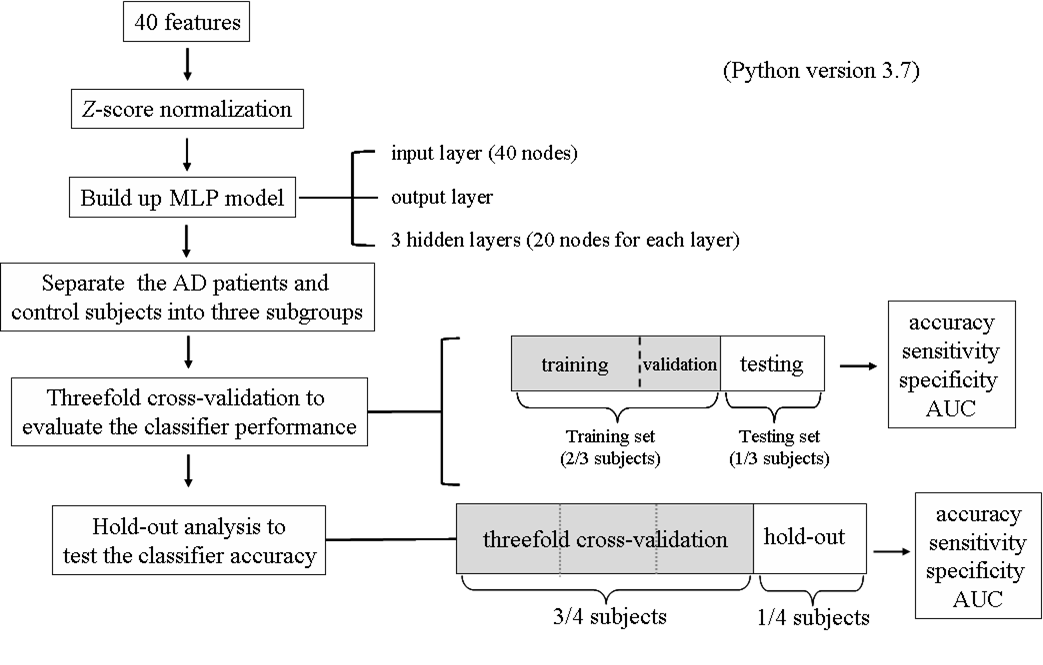


**MLP details (Jupyter 2.2.6)**

| Name | Version | Build_Channel |
| --- | --- | --- |
| scikit-learn | 0.21.3 | py37h6288b17_0 |
| seaborn | 0.9.0 | py37_0 |
| matplotlib | 3.1.1 | py37hc8f65d3_0 |
| more-itertools | 7.2.0 | py37_0 |
| numpy | 1.16.5 | py37h19fb1c0_0 |
| pandas | 0.25.1 | py37ha925a31_0 |
| tensorflow | 1.15.0 | mkl_py37h3789bd0_0 |
| keras-preprocessing | 1.1.0 | py_1 |

**Fig.S1.** MLP analysis results for comparisons of (a) BPW and (b) PPG indices between AD patients and control subjects.

Training and validation accuracy plots, AUC, and contradiction matrix are presented for the threefold cross-validation. “1” indicates AD patients and “0” indicates control subjects. For BPW indices, there were 6247 and 6626 pulses for AD patients and control subjects, respectively. The mean accuracy, sensitivity, specificity, and AUC were 89.83%, 81.00%, 98.67%, and 0.90, respectively. For PPG indices, there were 4673 and 4043 pulses for AD patients and control subjects, respectively. The mean accuracy, sensitivity, specificity, and AUC were 65.21%, 64.00%, 69.44%, and 0.65, respectively.

(a) BPW

(1) The training and validation accuracies were 96.13% and 98.53%, respectively, after 300 iterations. There were 1979 and 2095 pulses for AD patients and control subjects, respectively. The accuracy, sensitivity, specificity, and AUC were 90.00%, 80.00%, 100.00%, and 0.9, respectively.


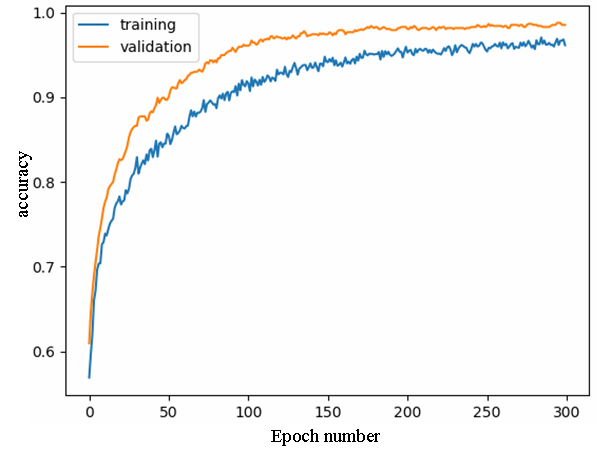

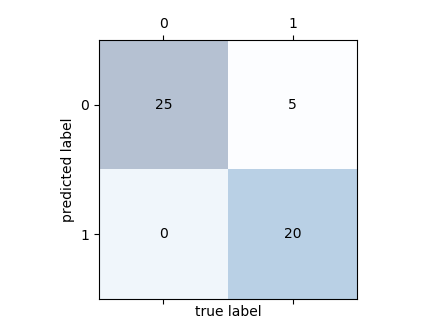


(2) The training and validation accuracies were 89.38% and 94.31% after 300 iterations. There were 2183 and 2210 pulses for AD patients and control subjects, respectively. The accuracy, sensitivity, specificity, and AUC were 92.00%, 88.00%, 96.00%, and 0.92, respectively.


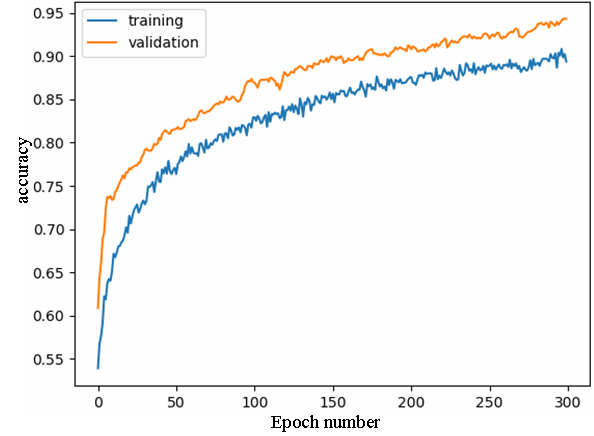

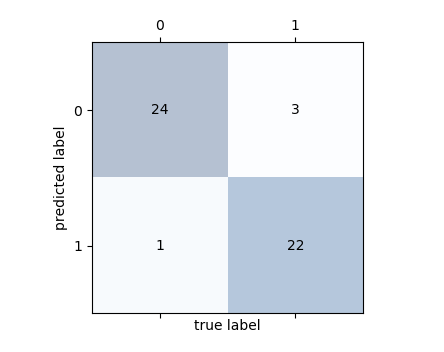


(3) The training and validation accuracies were 92.68% and 95.80% after 300 iterations. There were 2085 and 2321 pulses for AD patients and control subjects, respectively. The accuracy, sensitivity, specificity, and AUC were 87.50%, 75.00%, 100.00%, and 0.88, respectively.


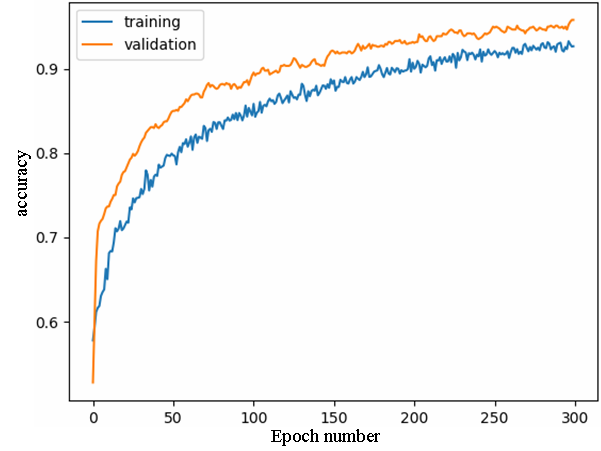

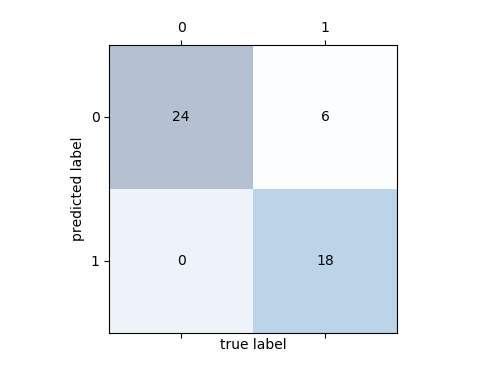


(b) PPG

(1) The training and validation accuracies were 85.19% and 87.13% after 300 iterations. There were 1349 and 1176 pulses for AD patients and control subjects, respectively. The accuracy, sensitivity, specificity, and AUC were 70.83%, 72.00%, 77.67%, and 0.71 respectively.


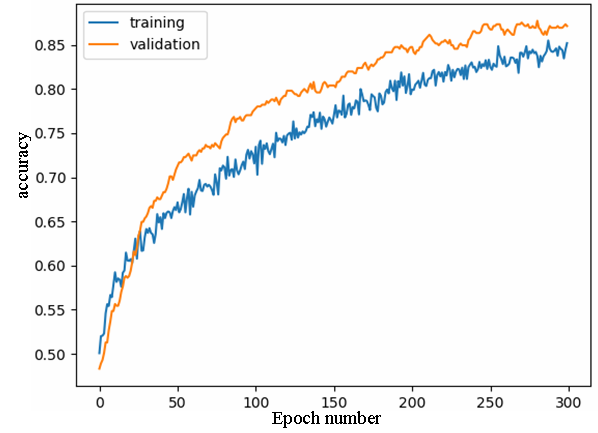

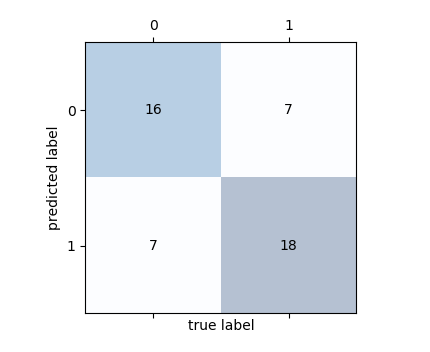


(2) The training and validation accuracies were 92.37% and 90.29% after 300 iterations. There were 1571 and 1315 pulses for AD patients and control subjects, respectively. The accuracy, sensitivity, specificity, and AUC were 63.83%, 50.00%, 78.26%, and 0.64, respectively.


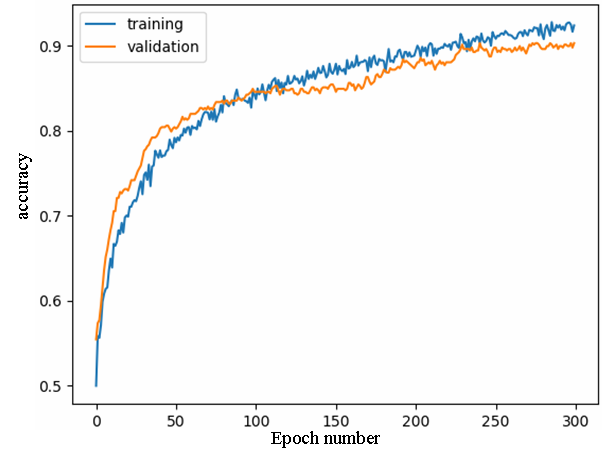

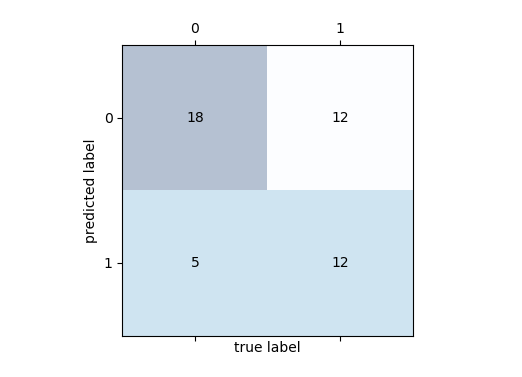


(3) The training and validation accuracies were 86.45% and 87.14% after 300 iterations. There were 1753 and 1552 pulses for AD patients and control subjects, respectively. The accuracy, sensitivity, specificity, and AUC were 60.98%, 70.00%, 52.38%, and 0.61, respectively.


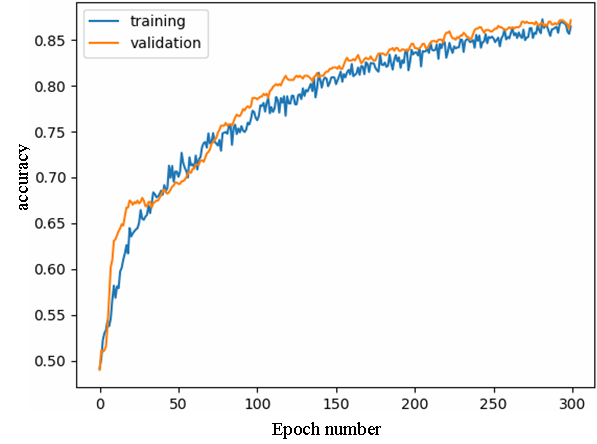

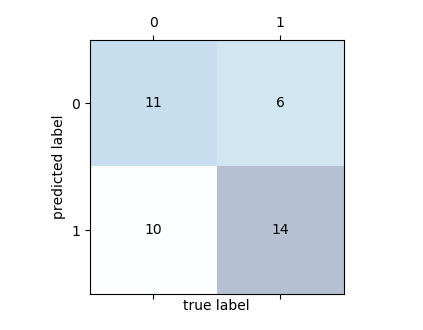


**Fig.S2.** Hold-out MLP analysis results for comparisons of BPW indices between AD patients and control subjects. (1)-(3): threefold cross validation; (4): hold-out test.

Training and validation accuracy plots, AUC, and contradiction matrix are presented. “1” indicates AD patients and “0” indicates control subjects. The mean accuracy, sensitivity, specificity, and AUC were 74.23%, 72.14%, 79.40%, and 0.74, respectively.

(1) The training and validation accuracies were 98.52% and 100%, respectively, after 300 iterations. There were 1508 and 1586 pulses for AD patients and control subjects, respectively. The accuracy, sensitivity, specificity, and AUC were 76.92%, 70.83%, 92.00%, and 0.77, respectively.

(2) The training and validation accuracies were 98.96% and 99.75% after 300 iterations. There were 1534 and 1633 pulses for AD patients and control subjects, respectively. The accuracy, sensitivity, specificity, and AUC were 76.32%, 75.00%, 77.78%, and 0.76, respectively.


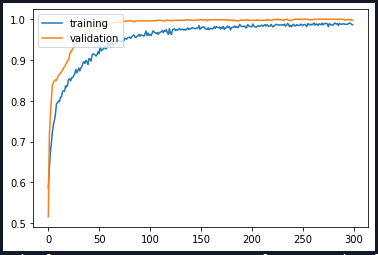

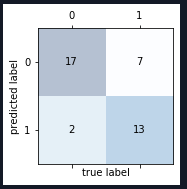

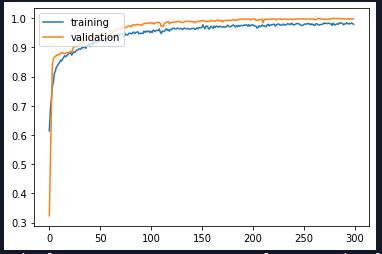

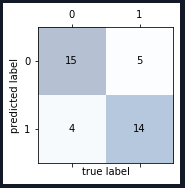


(3) The training and validation accuracies were 99.22% and 99.68% after 300 iterations. There were 1608 and 1705 pulses for AD patients and control subjects, respectively. The accuracy, sensitivity, specificity, and AUC were 69.44%, 70.59%, 48.42%, and 0.69, respectively.


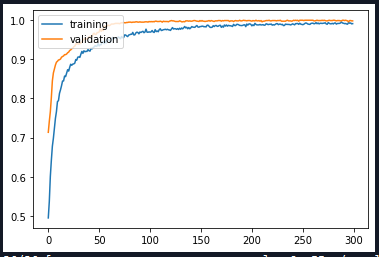

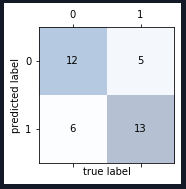


(4) The training and validation accuracies were 98.83% and 99.80% after 300 iterations. In the holdout, there were 1597 and 1702 pulses for AD patients and control subjects, respectively. The accuracy, sensitivity, specificity, and AUC were 82.86%, 77.72%, 92.31%, and 0.83, respectively.


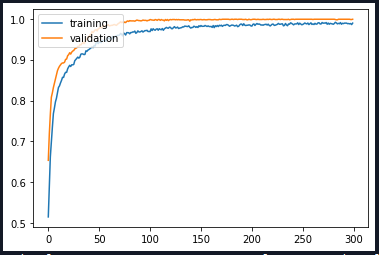

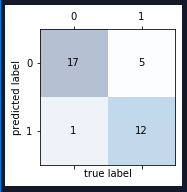


**Fig.S3.** MLP analysis results for comparisons of (a) BPW and (b) PPG indices between patients with mild and severe AD.

Training and validation accuracy plots, AUC, and contradiction matrix are presented for the threefold cross-validation. For BPW indices, there were 941 and 913 pulses for severe and mild AD, respectively. The mean accuracy, sensitivity, specificity, and AUC were 68.09%, 65.00%, 70.00%, and 0.68, respectively. For PPG indices, there were 637 and 634 pulses for severe and moderate AD, respectively. The mean accuracy, sensitivity, specificity, and AUC were 74.28%, 40.00%, 92.59%, and 0.66, respectively. “1” indicates severe AD and “0” indicates moderate AD.

(a) BPW

(1) The training and validation accuracies were 88.27% and 95.08% after 300 iterations. There were 307 and 302 pulses for severe and mild AD, respectively. The accuracy, sensitivity, specificity, and AUC were 66.67%, 60.00%, 70.00%, and 0.65, respectively.


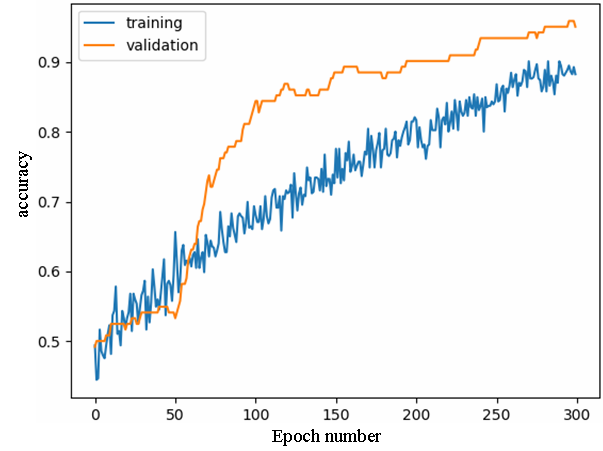

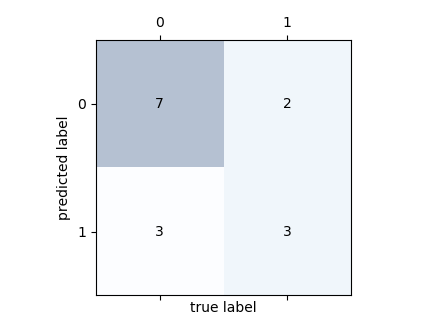


(2) The training and validation accuracies were 99.12% and 100.00% after 300 iterations. There were 348 and 334 pulses for severe and mild AD, respectively. The accuracy, sensitivity, specificity, and AUC were 64.30%, 75.00%, 60.00%, and 0.68, respectively.


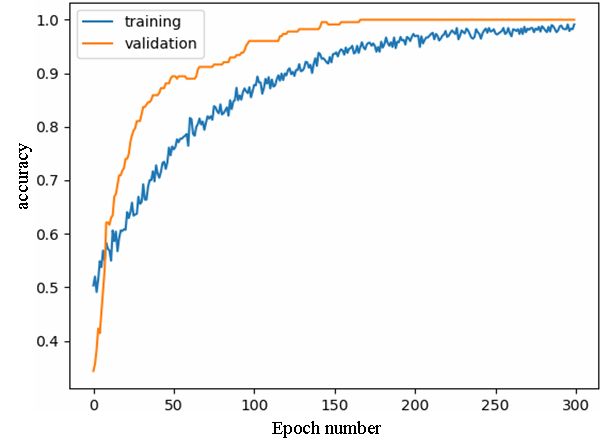

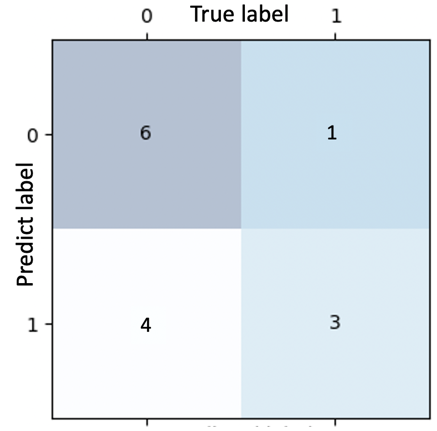


(3) The training and validation accuracies were 98.69% and 99.53% after 300 iterations. There were 286 and 277 pulses for severe and mild AD, respectively. The accuracy, sensitivity, specificity, and AUC were 73.30%, 60.00%, 80.00%, and 0.70, respectively.


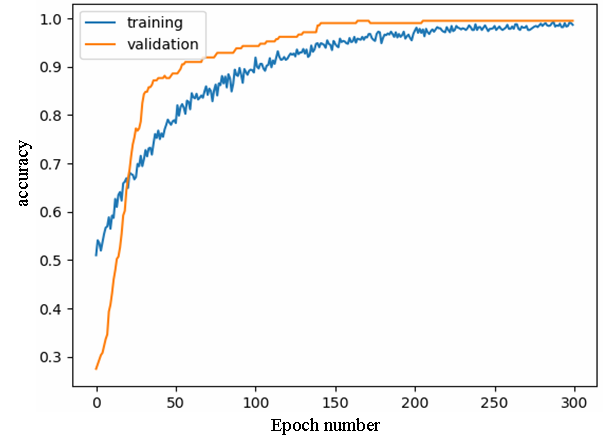

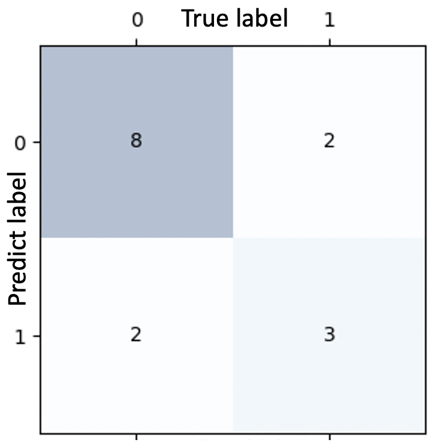


(b) PPG

(1) The training and validation accuracies were 82.19% and 82.72% after 300 iterations. There were 200 and 202 pulses for severe and moderate AD, respectively. The accuracy, sensitivity, specificity, and AUC were 80.00%, 40.00%, 100.00%, and 0.70, respectively.


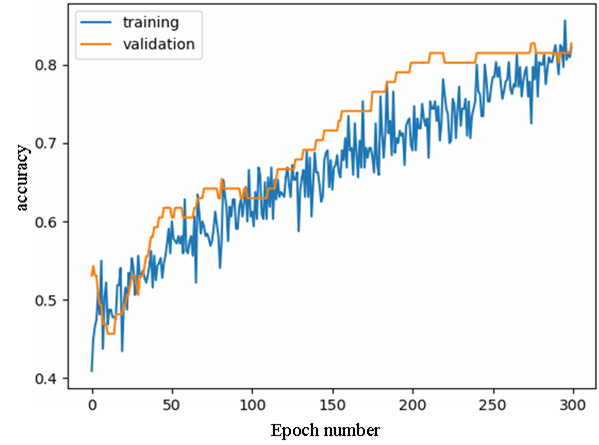

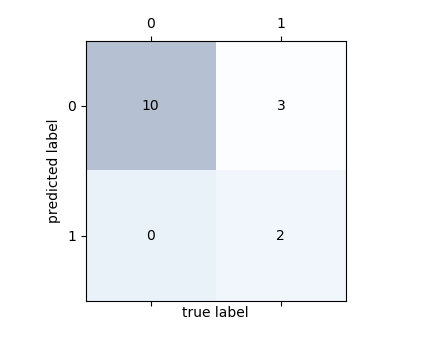


(2) The training and validation accuracies were 94.12% and 93.33% after 300 iterations. There were 226 and 222 pulses for severe and moderate AD, respectively. The accuracy, sensitivity, specificity, and AUC were 71.43%, 60.00%, 77.78%, and 0.69, respectively.


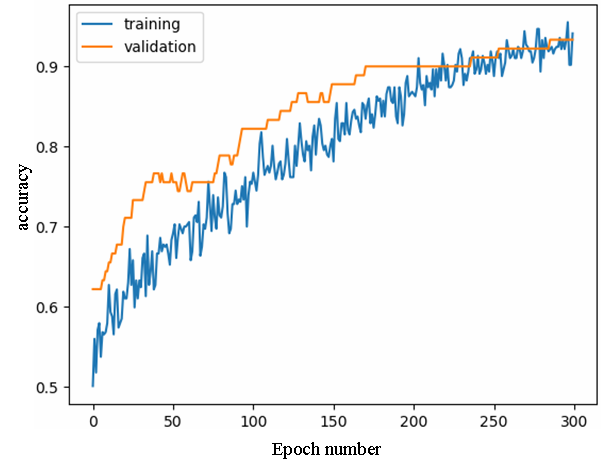

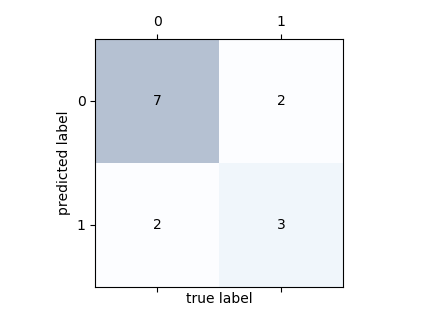


(3) The training and validation accuracies were 80.95% and 91.67% after 300 iterations. There were 211 and 210 pulses for severe and moderate AD, respectively. The accuracy, sensitivity, specificity, and AUC were 71.43%, 20.00%, 100.00%, and 0.60, respectively.


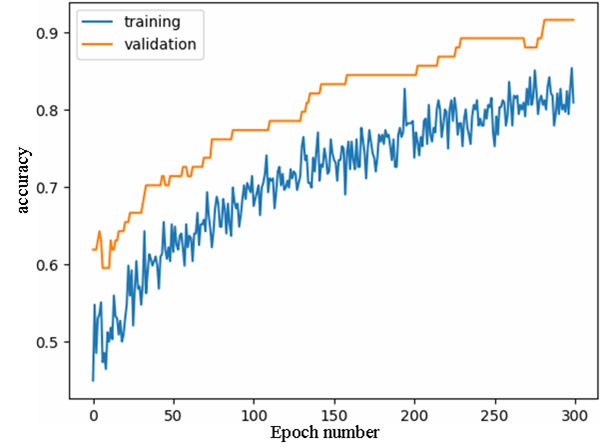

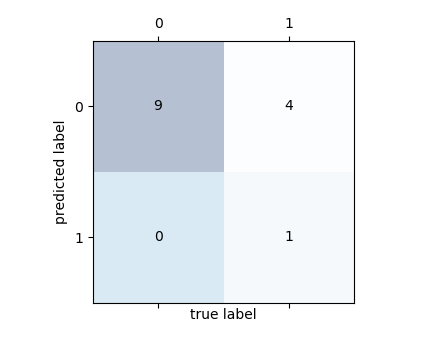

Supplement: Supplementary file 1 — Supplementary Information. [file 41598_2021_87903_MOESM1_ESM.doc]
